# Contextualized perspectives of well-being for adolescent girls: a qualitative metasynthesis

**DOI:** 10.1080/17482631.2021.1940766

**Published:** 2021-06-20

**Authors:** M. Isidora Bilbao-Nieva

**Affiliations:** Department of Psychology, Michigan State University, East Lansing, MI, USA

**Keywords:** Well-being, adolescents, metasynthesis, contextual, gender, adolescent girls

## Abstract

**Purpose**: This metasynthesis reviews and synthesizes the existing qualitative research on adolescent well-being, with explicit attention to how context shapes girls’ well-being. Well-being can be understood as a product of transactions between the individual and their context. Using qualitative research, this metasynthesis shows that girls face several threats to their well-being, often related to gender interwoven with the context in which girls operate.

**Methods**: A Qualitative Metasynthesis was used. It included 10 qualitative studies.

**Results**: Findings of this metasynthesis explain that context plays a relevant role in girls’ access to resources and opportunities within families and communities. They show that context defines expectations on how girls should behave and spend their time, which often become stressors for girls. Girls examine their context and use it to set up ideals and compare themselves to others. These comparisons impact girls’ self-image and structure aspects of their social life.

**Conclusions**: Context and gender affect girls’ well-being at different levels. Therefore, interventions and public policies should study the key factors girls identified as influencing their well-being, and address them using a multilevel rationale. Qualitative research can offer complex and illustrative insights on girls’ well-being, exploring their perspectives and experiences, and shedding light on how interlocking systems of oppression affect their well-being.

## Background

Within psychology and other disciplines, the study of well-being has emphasized that it is an umbrella concept that entails material and subjective elements (Gough & McGregor, 2007). As a construct related to the human experience, the experience of well-being is shaped differently, depending on factors such as gender, race, age, class, and culture (Camfield et al., 2009; Igarashi, 2015). Traditional approaches to well-being tend to be focused on individual levels, studying psychological constructs related to positive development (Seligman & Csikszentmihalyi, 2014), and the experience of positive affect and life satisfaction (Diener et al., 2017) as markers of well-being. These approaches often underestimate the effects of environmental factors in peoples’ well-being (White, 2017), including the impacts of context on how people evaluate and think about their own life (Fattore et al., 2019). On the contrary, other authors have emphasized the contextual nature of well–being, defining it as a collective state of affairs that emerges from positive relationships between people and the environment in which they operate (Evans & Prilleltensky, 2007) and as a phenomenon that should be understood from relational -and not individual- standpoints (White, 2017). These new definitions expand the notion of well-being beyond an individual level of analysis. Likewise, critical approaches to the study of well-being have stressed that the strategies to promote it should be focused on understanding and transforming systemic structures (Evans, 2014; Igarashi, 2015), and the achievement of social justice, as a condition that plays a key role on the achievement of well-being (Prilleltensky, 2012).

The study of well-being using qualitative methods gained relevance lately as a way to counterpart the quantitative knowledge, understanding the phenomenon of well-being from the standpoint of those who experience it, exploring their perspectives and opinions (González-Carrasco et al., 2019), and moving beyond traditional methods based on experts’ opinions (Newton & Ponting, 2013). Also, qualitative research is helpful to understand how different barriers to well-being are experienced intertwined with each other and affect individuals in multiple and complex ways (Wodon et al., 2018). Qualitative methods to study well-being recognize that notions and perspectives regarding well-being are social constructions tightly connected to the context in which they appear (Fattore et al., 2019). Authors using qualitative research propose that well-being should be studied linked to the social, cultural, and historical context in which well-being is manifest (Atkinson et al., 2017; White & Blackmore, 2015), avoiding generalizations of ideas of well-being.

The study of well-being as a contextualized phenomenon also has ethical reasons since it avoids cultural impositions of what constitutes a good life (White & Blackmore, 2015). In that line, qualitative researchers are committed to capturing and amplifying the voices of underrepresented groups and their perceptions of well-being (Camfield et al., 2009; Fattore et al., 2019; Savahl et al., 2015). From this perspective, qualitative research helps understand well-being in a contextualized way, exploring experiences and ideas of people in excluded social groups, and highlighting that their knowledge is fundamental information for its promotion.

In that line, some social groups have experienced disproportionate amounts of suffering and exclusion, and their well-being has been affected in multiple ways. Adolescent girls are recognized as one of these groups, as they face a myriad of injustices, forms of violence, and marginalization around the world. For example, at least 25% of adolescent girls ever-partnered experienced intimate partner violence, and about 13 million have experienced forced sex (UNICEF, UN Women & Plan International, 2020). The Global Women’s Institute at The George Washington University’s (2019) found that as of 2012, about 60 million girls were assaulted on their way to school every year and that in some countries, it was more probably to be sexually assaulted than became literate for a girl. The World Bank has shown that only 34% of girls complete their secondary school education in low-income countries (Wodon et al., 2018). And about 24% of adolescent girls around the world are neither in education, employment, or training (UNICEF, UN Women & Plan International, 2020). In addition, it is essential to distinguish between the experiences of different groups of adolescent girls around the world to delve into the diversity of threats to well-being they face. Such diversity is also present in the way adolescent girls understand their well-being, which represents an important contribution to the field, as it moves beyond dominant narratives that have positioned some social groups and their ideas as the norm (Igarashi, 2015).

Grounded in feminism, this qualitative metasynthesis rejects essentialist assumptions of female adolescents and their well-being. Feminist theorists in psychology have advocated for researchers to recognize that gender differences exist and need to be identified and studied, illuminating the androcentrism pervasive in the discipline (Cosgrove, 2003). Among those, for example, Jean Baker Miller (1976) proposed that specific psychological ways of functioning are common for all women. However, she also has purposely erased from her analysis the racial and class-based factors that determine radical differences in women’s experiences (Miller, 1976). Similarly, Carol Gilligan developed a women-centred theory of morality; however, this theory failed to acknowledge the experiences of other women outside of the white, middle-class position (Wigginton & Lafrance, 2019).

Thus, although these researchers have advanced feminism within psychology, they have also been accused of essentialism, as they promote one point of view that claims to represent all women and womanhood (and girls and girlhood), locating their processes and experiences from an individual, intra-psychic focus (Cosgrove, 2003), and ignoring the undeniable differences made by a multiplicity of intersecting social contexts that shapes people’s lives.

In this metasynthesis, a different feminist approach is adopted to acknowledge the forces of the environment involved in the production of the individual itself (Cosgrove, 2003). Therefore, ideas of gender-based normativity through trying to establish theories that point out a “women’s relational style” (Cosgrove, 2003) are contested and avoided. In that sense, this metasynthesis advocates for a non-essentialist perspective that centres on changing the structural rather than the individual.

In this metasynthesis, well-being is understood as an experience that cannot be separated from the context in which it emerges. It aims to elucidate **how adolescent girls’ well-being is shaped by their interactions with their context**. In this metasynthesis, gender is considered to play a key role in the way adolescent girls perceive and imagine having a good life and as determining spaces of exclusion and threats to well-being. Across disciplines -psychology included- feminist researchers have assumed the task of revising scientific knowledge, often written from the perspectives of educated white males (Wigginton & Lafrance, 2019). This metasynthesis builds upon the current qualitative research on adolescent well-being, capturing singularities of female participants and their experience. It is an assumption of this metasynthesis that qualitative research can provide important insight regarding the contextual factors that affect girls’ well-being on different cultural contexts. Therefore, it advances the study of adolescent well-being by illuminating the experience of female adolescents and their relationship with the context in which they operate. This is done from a feminist perspective that allows the possibility of challenging hegemonic and patriarchal discourses as they have material consequences in girls’ lives, and determine gendered patterns of social relations (Mattos, 2015).

## Methods

### Design

The current metasynthesis uses the approach proposed by Sandelowski and Barroso (2007). According to these authors, a qualitative metasynthesis comprehends a systematic revision of the current state of qualitative literature and generates new knowledge through an interpretative synthesis of the aggregated data. This interpretative step goes beyond aggregating data.

Qualitative metasynthesis emerges as a response to the underutilization of the knowledge produced by qualitative studies (Sandelowski & Barroso, 2007). This approach opens the possibility of creating an interpretative dialogue between studies to reach an enriched and more complex comprehension of a social phenomenon (Thorne, 2017). The dialogue is nourished by the different disciplines and angles of inquiry that illuminate different aspects of the same research object (Thorne, 2017).

Considering the multiplicity of theoretical and methodological approaches to the study of well-being, a qualitative metasynthesis is a good method to synthesis the qualitative literature without reducing its complexity and without subordinate some articles to others. In this case, the qualitative literature on adolescent well-being is reviewed and harnessed to shed light on a new analytical question related to the female adolescent experience of well-being.

### Search and selection strategy

Searches on English, Spanish, and Portuguese were done using ProQuest and Scielo. Since the qualitative literature on well-being is scarce in comparison with the quantitative, the search was done without restrictions on time frames. The SPIDER tool was used to set the inclusion parameters (Cooke et al., 2012; Methley et al., 2014). This tool is designed specifically for reviewing empirical qualitative data, enhancing search precision and efficiency (Cooke et al., 2012). Theoretical articles were excluded from the revision. The parameter definitions using the SPIDER tool were: **S**ample (adolescents) **P**henomenon of **I**nterest (well-being) **D**esign (qualitative data collection and analysis) **E**valuation (experiences and perceptions) **R**esearch type (Qualitative methods).

**Figure 1.**
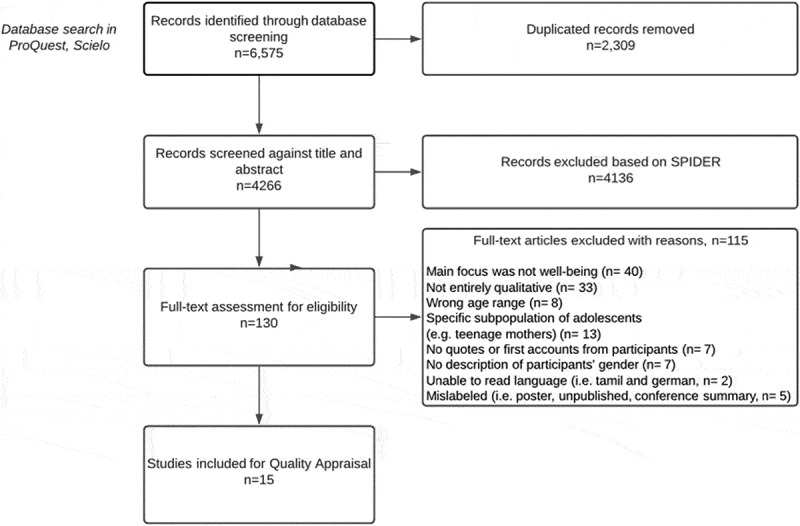
Phases of screening

The search conducted on ProQuest uses the following terms: (well-being OR well-being OR “life satisfaction” OR “quality of life”) AND ab(adolescent* OR teenager OR “young people”), AND (qualitative), ab(qualitative OR ethnography OR “grounded theory” OR “thematic analysis” OR narrative OR phenomenology OR “content analysis” OR “discourse analysis”). Then, a new search was conducted using Scielo to find literature in Spanish and Portuguese, with the same terms translated. Results were exported to Covidence, a software for systematic reviews. After duplicate removal and further revisions using the SPIDER tool, 16 documents were retrieved for appraise quality (two dissertations, one working paper, and 13 peer-reviewed articles). Figure 1 shows the phases followed for article screening and selection.

### Quality appraisal

The quality appraisal was done using guidelines from the Critical Appraisal Skills checklist for qualitative research (Critical Appraisal Skills Programme, 2018). This is a comprehensive tool, recommendable for qualitative metasynthesis assessments (Finfgeld-Connett, 2018). It includes ten questions related to the strength of the study design, quality, and clarity of their findings and their relevance. Each article was assessed to determine its inclusion, and the appraisal was written on separate memos to ensure the process was clear and justified. Based on this appraisal, five articles were excluded from the metasynthesis due to their lack of clarity or methodological consistency. Two of the articles were excluded because they presented only descriptive results. One of these presented the results as a list of quotes without analysing them, and the other one based its analysis on repeated words. One article used a purposive sampling that only included participants from one specific population -first generation of Latinas living in the U.S.-, which largely affected the results. Another article reported its qualitative results merged with previous quantitative research. Finally, an article that organized its results according to a different variable (not well-being) was also excluded at this stage.

### Analytical strategy

After the final selection was over, the studies were analysed individually, and one analytical memo was written for each one. In these memos, three major sections were included: 1) analysis of the theoretical approach to well-being, 2) analysis of the reported context, and 3) analysis of the approach to gender. Then, these individual memos were reviewed, compared, and synthesized into a general analytical memo, in which common elements, differences, and general interpretations were included. A separate general methodological memo was also done, including comments on ethical aspects and impacts of the methods in the results. The analytical and methodological memos were used to write the results section. This analytic strategy was designed based on the recommendations of several qualitative researchers that support the use of memos to organize the analysis and encourage reflexivity (Saldaña, 2013), as well as other analytic strategies that involve memos, described in other metasynthesis (Molony, 2010).

## Results

The first finding of this metasynthesis is that the qualitative literature on adolescent girls’ well-being is still very scarce. The total amount of studies included in this metasynthesis (9 peer-reviewed articles and one Ph.D. dissertation) is within the typical range for this kind of revision (Finfgeld, 2003). However, the number of studies exclusively dedicated to examining girls’ well-being was very small (5). The paucity of research dedicated to girls’ well-being makes apparent the need to produce more knowledge regarding their well-being experiences, adding complexity and diversity to the study field.

Table I summarizes the general information about the included studies. Demographic characteristics are described below, while the theoretical orientation to well-being is discussed in a separated sub-section.

**Table I.** Characteristics of included studies

No.	Author, year of publication.	Sample (Female)	Country of study	Age	Methodology	Method of data collection	Well-being theoretical approach
	Helseth and Misvær (2010)	31 (20)	Norway	14–15	Phenomenology	Individual interviews	Psychological well-being; complementary to health.
	Larsson et al. (2013)	15 (15)	Sweden	13–19	Phenomenology	Individual interviews	Synonym of Health
	Wiens et al. (2014)	117 (117)	Finland	13–16	Descriptive qualitative research	Electronic open-ended questionnaire	Psychological well-being
	Flacking et al. (2014)	18 (18)	Sweden	17–18	Grounded Theory	Individual interviews	Psychological well-being
	Glozah (2015)	11 (6)	Ghana	16 (ave)	Thematic analysis	Individual interviews	Psychological well-being; Synonym of Health
	Phiri and Abebe (2016)	21 (12)	Zambia	9–15	Ethnography	Interviews, focus groups, fieldwork observations	Subjective/Relational/ Contextual/Processual well-being
	Wilson and Somhlaba (2016)	18 (8)	Ghana	17–22	Thematic analysis	Individual interviews	Subjective well-being; Life satisfaction
	Wiens et al. (2017)	117 (117)	Finland	13–16	Descriptive qualitative research	Electronic open-ended questionnaire	Psychological Well-being
	Bintliff (2019)	10 (10)	USA	12–13	Grounded theory	Group interviews, journal entries, visual art.	Subjective Well-being; Life satisfaction.
	Vujčić et al. (2019)	14 (unknown)	Croatia	13–15	Qualitative framework analysis	Individual interviews, focus groups.	Psychological well-being; Subjective well-being.

As this table shows, the selected studies were conducted in Sub-Saharan Africa (Ghana and Zambia) and Nordic Countries (Finland, Norway, and Sweden). There was one study conducted in the U.S. (Ph.D. dissertation) and another one in Croatia. Considering the U.S. as a country with an important tradition in social science research, it was surprising that the production of qualitative studies in adolescents’ well-being was so limited. In the same vein, it was also unanticipated that other countries of Europe were not present in the selection.

The adopted designs were phenomenology (2), grounded theory (2), ethnography, and versions of descriptive qualitative analysis (thematic analysis, qualitative content analysis). The studies using phenomenological approaches aimed to understand how well-being was experienced from an adolescent perspective. The ethnographic study was focused on understanding how adolescents’ well-being was interwoven with the social practices of a particular community. Grounded theory was used as a way to organize different elements that participate in adolescents’ well-being definitions, and descriptive qualitative analysis was used to identify recurrent elements that contribute to or impede well-being.

All the studies selected were in English. Although studies in Portuguese and Spanish were evaluated for their inclusion, unfortunately, they did not meet the selection criteria. Sample sizes vary from 10 to 117. Traditionally, many variants of qualitative research adopt small sample sizes. In that sense, the only sample size that stands out is Wiens et al. (2014, 2017), that reported a study with 117 participants. This sample size is justified in the article by the methodological design adopted (descriptive qualitative analysis) that includes collecting small amounts of written data from several participants. According to the authors, the data collection strategy is also considered a weakness of the study design, as the short-written answers were ambiguous in several cases.

### Orientation to well-being

The selected studies are not agreed upon their definition or operationalization of well-being. Two major approaches are distinguished among these definitions: from psychological perspectives (subjective well-being or psychological well-being) and from a health perspective.

Regarding psychological perspectives, several of the selected works adopted an orientation to the study of well-being as trying to understand adolescents’ perceptions of satisfaction with life and evaluation regarding what and how different types of factors influence their subjective well-being (Bintliff, 2019; Flacking et al., 2014; Helseth & Misvær, 2010; Larsson et al., 2013; Vujčić et al., 2019; Wiens et al., 2014, 2017; Wilson & Somhlaba, 2016). These articles build knowledge on contextual issues recognized by the adolescents as impacting their individual life evaluations on a daily basis. The study of preferences, subjective evaluations, and perceptions of satisfaction with life are often circumscribed in psychological traditions -e.g., Diener et al. (2017)—and the use of this research has a particular emphasis on promoting elements that are highlighted by adolescents as important for their well-being, like family relationships and social support.

Sometimes in combination with the above, a second adopted perspective to well-being was as related to, complementary to, or used as a synonym of health (Glozah, 2015; Helseth & Misvær, 2010; Larsson et al., 2013; Wiens et al., 2014, 2017; Wilson & Somhlaba, 2016). In those articles, well-being is approached as an overarching concept that included physical and mental health and advanced beyond the absence of illness, in line with the conceptual framework of the World Health Organization. Almost all of these studies are published in the field of health studies and nursing. Context is considered relevant by the authors when they represent different forms of threats to adolescents’ health, and its promotion was centred on developing healthy behaviours.

On a different line, Phiri and Abebe (2016) focus their study on ideas of well-being that derive from shared patterns of what is considered valuable for adolescents within a community. In their study, they explain how context, understood as certain daily social practices, is connected to the way people describe and understand what constitutes a good life. In this article, Phiri and Abebe (2016) invite researchers to theorize and study well-being as a concept that is always context-specific and emphasize that well-being results from the interplay between subjective evaluations and objective circumstances in which people operate.

Similar to the above, Glozah (2015) studies the concept of well-being as socially constructed and as a result of social influences and community variables (i.e., social support) that impact the well-being of the overall community. This understanding of well-being positioning it as a shared community phenomenon instead of the traditional individual approach.

As mentioned before, the question that guided this analysis was **how adolescent girls’ well-being is shaped by their interactions with their context**. To answer that question, the findings of this metasynthesis are organized into two sections. First, the role of context and gender in the selected studies is reviewed, serving as a framework for the second section. In the second section, three ways in which context shapes girls’ well-being are described: 1) context defines unequal access to resources and opportunities, 2) context sets expectations of how girls should behave and spend their time, and 3) girls examine and use context to set ideals and produce comparisons with other women and girls.

## Orientation to context and gender

The studies adopted different approaches to what constitutes context, as well as concede different levels of importance to gender in their results and analysis. These differences largely influenced the results of this metasynthesis, as they determine the possible interpretations of how well-being was shaped by the context and how gender was playing a role in that process.

### Orientation to context

Context and its influence can be understood at different levels or scales. However, the majority of the articles selected are focused on a microsystemic level, particularly on types of interaction with family and peers, living arrangements, and school experience. For example, these studies pay attention to girls’ attachment styles with parents, and their influence on their psychological well-being (Flacking et al., 2014), how different types of friendships and peer-relationships affect participants’ quality of life (Helseth & Misvær, 2010), family socioeconomic status and living infrastructure and its influence on what is understood as well-being (Phiri & Abebe, 2016), and the impact of family stressors on subjective well-being (Bintliff, 2019). The importance conceded to microsystemic levels on the experience of well-being is highlighted by Larsson et al. (2013) and by Phiri and Abebe (2016), who propose well-being is a daily life experience constrained by family and community.

Different from the other articles, Wilson and Somhlaba (2016) identified as relevant context not the most direct microsystem, but the larger community setting in which the participants live, including material resources and cultural factors. They dedicated a section of their article to explain Ghana’s sociohistorical context using it as a background to present their research. In this article, the authors highlight an overall community context of adversity and impoverishment and link how those conditions have an effect on the development of a particular idea of well-being within the community as a whole. This important analytical difference is reflected in results, focused on contextual factors and their role in the emergence of psychological well-being within a community.

The studies also present different views regarding the extent to which context can be changed. In some of the articles, context is taken as a set of fixed conditions, and well-being is related to the possibility of adolescents to maintain hope, resilience, and be realistic in their expectations (Vujčić et al., 2019; Wilson & Somhlaba, 2016) or strengthen psychological resources to face adversity (Bintliff, 2019). In those cases, well-being depends on individual abilities to tolerate contextual hardships, and it is most probably achievable by strengthening individuals instead of changing living conditions. These approaches to well-being also determine what types of recommendations for well-being promotion are presented in the studies, often related to helping adolescents in the development of these psychological resources. On the contrary, Phiri and Abebe (2016) emphasize that through focusing on individual responses to contextual adversity, the study of well-being has de-politized the context. From their perspectives, the structural and social forces that impact well-being should be examined and ultimately modified, addressing in that way relevant causes of individual ill-being.

### Orientation to gender

Regarding the role of gender, as mentioned before, 5 of the 10 reviewed studies had girls-only samples (Bintliff, 2019; Flacking et al., 2014; Larsson et al., 2013; Wiens et al., 2014, 2017). The justification for only including girls was that they report levels of health and satisfaction with life that were lower than boys. Despite this consideration, the results were often reported without explicitly addressing gender singularities or making analytical connections between gender and the research questions of the study. In addition, these researches did not expose reasons or explanations to explain why girls would have lower levels of life satisfaction or health.

Similarly, the articles that included adolescents of all genders reported scarce information regarding gender differences or particularities. Within the exceptions, there is an explicit comment regarding sociocultural barriers for girls’ well-being in the article written by Wilson and Somhlaba (2016), and some comparisons of factors contributing to well-being highlighting differences between girls and boys in Phiri and Abebe (2016). Excluding these exceptions, the vast majority of findings on the selected studies were stated from a gender-neutral standpoint. For example, in Glozah (2015), there is only one comment related to the gender of the participants. In consequence, the results of the studies are described as homogeneous for all adolescents, disregarding to report and examine the particular impact of gender in the living experience of well-being, as a relevant analytical category for the study of well-being in qualitative literature.

Despite the paucity of a gender perspective in the selected studies, the interpretation of this metasynthesis was focused on making gender particularities visible. The interpretative lens adopted by this metasynthesis was handy for this purpose, with special care of not overinterpreting results or report evidence that was not present in the original studies. Most of these particularities were noticeable in specific barriers to girls’ well-being and how girls shape their ideas and experience of well-being linked to their gender and context.

## Context and gender and their influence on well-being

### Unequal access to resources and opportunities

Adolescents recognized the importance of counting with basic material resources such as food, clothes, or appropriate housing (Glozah, 2015; Phiri & Abebe, 2016; Wiens et al., 2014; Wilson & Somhlaba, 2016). Some of the analysed studies highlighted that girls have less access to resources and opportunities than boys in several dimensions of their lives and are disproportionally affected by contexts of adversity, violence, and poverty (Glozah, 2015; Phiri & Abebe, 2016; Wilson & Somhlaba, 2016). These inequalities determine specific barriers to their well-being, as well as permeate girls’ ideas of what is critical for their well-being and how well-being is experienced by girls.

Some of the studies described specific patterns within families and communities that can be interpreted as naturalized ways to reproduce gender inequality within communities. Wilson and Somhlaba (2016), for example, show that due to cultural norms of early marriage in Ghana, girls often do not receive education. Families in this society understand girls’ education as futile, while boys in the same family can have their access to education warrantied. Depending on the resources and family will, girls would keep receiving education or would move to their husbands’ homes and abandon school. Within this context, the authors describe that girls have normalized this exclusion, understanding the possibility of receiving education as a privilege and not as a right, perpetuating, therefore, cycles of disadvantage and feminization of poverty.

In Phiri and Abebe (2016), the authors found that girls are more pressured by their families to do chores at home and also to contribute to the family livelihood, which means high levels of exhaustion that have detrimental effects on their well-being. The everyday experience of these girls is affected by normalized inequalities on the distribution of responsibility. This exhaustion also has effects on the way girls understand well-being. Girls in this study described the importance of rest as a critical condition for well-being. Although being able to rest as critical for well-being was mentioned in other studies too (Flacking et al., 2014; Glozah, 2015; Wiens et al., 2017), in this case it was explicitly and closely related to a context in which girls endure exhaustive activities. Therefore, high levels of exhaustion made girls value and explicitly identify rest as a fundamental condition for well-being.

Likewise, Bintliff (2019) describes girls’ experience as affected by a context that privilege men over women. The author found that girls see themselves as lacking freedom and safety in their immediate environment. They fear being kidnapped when walking on the streets, being raped or sexually assaulted, and not feeling protected by others. The author also explained that some of the participants had been sexually harassed by peers at school. In her analysis, Bintliff state the social context is misogynistic, stressing that boys face very few consequences if they make their female peers feel unsafe. In this study, girls operate in an environment in which these hazards to well-being are experienced daily.

In addition, gender plays an important role in the way environmental conditions are experienced by adolescents. The studies showed that some conditions become relevant for girls and not to for boys, becoming critical for their well-being. For example, also in Phiri and Abebe (2016), gender-based differences are explicit in material and infrastructure elements that become fundamental for girls’ well-being. In their study, the authors highlight differences between girls and boys, explaining that girls concede specific importance to domestic items as markers of well-being since they are needed for them to meet specific roles within their households. These preferences show that well-being is an experience that derives from daily social practices circumscribed in a specific context. In this case, household chores assumed by girls as normalized social practices define material preferences and, therefore, scarcities that affect girls and not boys.

In the same line, these authors also identify that the concrete condition of not having a toilet at home was particularly relevant for adolescent girls. This was interpreted by the authors as connected to specific threats to safety that affects girls over boys. Girls that do not have toilets at home are forced to use open spaces, which expose them to sexual and hygienic dangers—having a toilet at home spares them from that risk, causing an important impact on their daily experience and becoming an essential condition for girls’ well-being.

### Expectations and contextual stressors

The studies showed that girls aspire to meet the expectations and exigences set by their communities. These expectations are derived from images of how a girl should behave and relate to other people. When these expectations were too exigent, or girls are unable to meet them, they become stressors and sources of ill-being.

Meeting expectations was connected to girls’ well-being in several of the studies. For example, in Larsson et al. (2013), the authors found that girls were very committed to assume and fulfill the requirements of the context. As the authors describe, through meeting community and family demands, girls in this study feel control over their own life and balance between commitments and leisure activities. These are deemed as important sources of well-being. In Phiri and Abebe (2016), girls connect well-being with an overall family harmony that derives from meeting their role at home. As this study showed, girls identify their specific role at home and associate their well-being with being able to meet that role, fulfiling family and community expectations.

Similar results were reported by Glozah (2015), who described adolescents’ feelings of well-being when they are able to accomplish their commitments and expected activities. Glozah’s results are not applied only to female adolescents, although it can be presumed that those commitments and expected activities are different depending on gender.

On the other hand, researchers also found that when these expectations become stressors mean important obstacles for girls’ well-being. For Wiens et al. (2014, 2017) describe girls feel good with themselves when they are able to meet academic expectations, but also feel stressed and pressured by their schools for academic achievement. In Wiens et al. (2017), the authors describe girls stressed by not being able to satisfy other people or disappointing parents and friends.

Other articles also highlight that girls feel pressured and stressed by the expectations of the environment. In Vujčić et al. (2019), the authors found that participants felt compelled to spending their time in certain activities and choosing certain career paths over others, which is interpreted as stressful social pressure. The authors use girls’ examples to make a distinction between adolescents’ wishes (i.e., careers in humanities and social sciences) and pressures of parents and society in general, related in this case to future financial success. Similarly, Larsson et al. (2013) also describe girls as being often overwhelmed and stressed by the numerous requirements of the context. In this context, the authors found that the inability to incorporate the demands of the context was connected to feelings of incapability, inadequacy, and exclusion from what is considered regular social life. Flacking et al. (2014) describe very similar feelings derived from stress, and shame as an important negative outcome of not being able to meet expectations. And in the study conducted by Bintliff (2019), the author found that girls unable to meet parental expectations also feel stressed and depressed.

In Phiri and Abebe (2016), the authors found that girls were expected to fulfill certain activities within their households related to their gender-based roles in their communities. Within the activities reserved for girls, hard chores such as fixing the roofs, or needing to walk long distances to fetch water for their houses, are considered by girls as exhausting, becoming stressful, and affecting their everyday experience at home. This was a clear example of how family and community expectations were often gender-based.

In addition, expectations of context were likely particularly stressful for girls over boys. For example, in the study conducted by Glozah (2015), the author reports adolescent girls were more stressed than boys, which was manifest in their seeking more help and support from significant others. Sources of ill-being and stress for adolescents in this study were related to strictness and pressure from school and parents, including not being heard in their opinions by family members, being ignored, and not having autonomy. These same sources of ill-being are presented in the study of Bintliff (2019) and in Flacking et al. (2014), both of which had a girls-only sample.

### Comparisons and ideals

Girls examine and compare their lives with the lives of other girls and women living in the community and use this comparison to define what is having a good life for themselves. They also evaluate themselves and their lives in relation to other girls, which in turn serves to determine their levels of well-being in relation to other girls.

To illustrate this point, in the analysis of Helseth and Misvær (2010) and in Flacking et al. (2014), the authors found that girls live in a very exigent social hierarchy, build on what girls look like, their level of attractiveness, and the number of friends they have. Comparing themselves with peers give girls a relative place on the social hierarchy. As Helseth and Misvær (2010) proposed, depending on their place within this social hierarchy, girls experience higher or lower levels of well-being. In this case, less popular girls had poorer self-images and, according to this study, lower levels of psychological well-being.

In Flacking et al. (2014), the authors found that descriptions of being attractive among girls were highly homogeneous. Girls named certain attributes to describe images of ideal, including physical and behavioural elements, and then use them to compare themselves to that ideal. According to these authors, peer comparisons can also create ill-being, serving as mediators of anxiety and even depression. Similarly, in both Larsson et al. (2013) and Bintliff (2019), girls compare themselves with others to set ideals and evaluate themselves and the way they look, determining levels of self-esteem which have a direct influence on their levels of well-being. According to Larsson et al. (2013) compare themselves with their peers and seek for similar appearance, using cosmetics and clothes as markers of a shared identity and belonging. On the other hand, Bintliff (2019) found that girls compare themselves to context-specific ideas promoted by media and feel compelled by the context to maintain comparative thinking regarding their body image. In this study, the author highlights that these ideas mean important sources of ill-being for girls.

These comparisons are also made regarding opportunities and material resources. For example, in Wilson and Somhlaba (2016), as mentioned before, authors showed that due to cultural norms of early marriage, girls often do not receive education. These authors explain that for those girls that see themselves in the position of being able to go to school, the possibility of receiving education becomes critical for their well-being and is interpreted as a sign of support from parents and a reflection of having a life that is better than some of their peers. Thus, by comparing themselves to more unprivileged peers, girls in this study evaluate their own lives and their relationship with parents, describing these are factors that positively influence their life satisfaction and define supportive parents for them.

## Discussion

As this metasynthesis showed, qualitative research on girls’ well-being can shed light on unique factors of the context that affect girls’ well-being. Several quantitative studies around the world have shown that girls’ health and well-being decline during adolescence years, although explanations of why this happens are still speculative (Skrzypiec & Askell-Williams, 2018). In addition, there is contested evidence regarding the relationship between gender and subjective well-being, which is interpreted by researchers as illustrating the complex interactions between gender, context, and culture (Casas et al., 2013; Montserrat et al., 2015). These results show the importance of qualitative research to study this population and how it can illuminate key aspects and mechanisms that influence female experiences of well-being.

The studies discussed in this metasynthesis shown the importance of immediate context for girls to experience well-being particularly focused on forms of microsystemic forces that have effects on girls’ daily lives. The belief that well-being is a daily living experience largely defined by family and community (Larsson et al., 2013; Phiri & Abebe, 2016) was prevalent in the selected studies. Interestingly, Phiri and Abebe (2016) propose that because family and community play such a relevant role in adolescents’ well-being, public policy efforts should be focused on those levels instead of directly to adolescents, moving the approach to well-being promotion beyond individual levels, and offer a more ecological view of the phenomenon. As mentioned before, the promotion of well-being only at individual levels have important ethical implications, since it makes individual responsible for their well-being, disregarding the fundamental influence of context in well-being distribution (Evans, 2014; White, 2017). For example, to promote girls’ well-being, schools and social programmes have focused their efforts on strengthening girls’ self-esteem, which is a remedial strategy that neglects structural levels of gender inequality (Wright & Mcleod, 2015).

In line with the above, the studies included in this metasynthesis clearly shown that gender norms played an important role in the way girls’ well-being was shaped by context. The evidence has stated that, around the world, gender norms have a major and undeniable detrimental influence on adolescents’ well-being (Levy et al., 2020). The same study showed that the majority of interventions devoted to promoting gender equality for well-being and health are targeted at individual behaviour and attitude change, neglecting to intervene on wider levels of the social structure (Levy et al., 2020). Without transforming cultural and societal forces that have determined gender norms, efforts to promote girls’ well-being are less likely to succeed. On that line, delving into understanding the mechanism by which gender norms affect girls’ well-being represents valuable knowledge that should be further explored.

Among the ways gender norms affect girls’ well-being is that they have an important influence on the way resources and opportunities are distributed within families and communities. Likewise, gender roles are combined with culture and patterns of intergenerational relationships that determine such distribution. Quantitative studies of poverty and well-being have presented important evidence regarding age as a factor that affects resource distribution within the family, finding that often children and adolescents have fewer material resources (Gross-Manos & Ben-Arieh, 2017; Yin Nei Cho, 2018). These studies emphasize the need to gather information regarding the way resources are allocated and distributed among different family members. They propose to study family dynamics to understand the underlying causes of these inequalities in the study of material poverty and well-being. According to the findings of this metasynthesis, the examination of these internal dynamics of resource distribution should also consider gender as a critical aspect since it evidently influences the patterns of female impoverishment within families and communities.

This metasynthesis also found that girls find important sources of well-being though fulfiling community and family expectations, and on the contrary, not being able to meet the expectations becomes stressors that yield more ill-being. In line with these results, quantitative studies with samples from different countries have shown that (1) girls experience more stressors than boys, and (2) also internalize them more, particularly those related to the interactions with friends and family, being their well-being more affected by these interactions (Flook, 2011). In addition, international studies have also shown that demands and expectations of society increase during adolescence (Ronen et al., 2016), suggesting that during adolescence, girls face more stressors. The results of this metasynthesis are corresponding with this evidence, as they show that girls are particularly sensitive to environmental stressors derived from family and community expectations. Furthermore, they supplement this knowledge by showing how the stress caused by the inability to meet expectations engenders feelings of exclusion from family and community, affects family harmony, and has impacts on girls’ self-esteem, linking how these different factors interact with girls’ well-being. In addition, it can be interpreted that meeting expectations serves as an important mechanism to maintain social practices and gender-based roles that may be abusive or perpetuate inequalities and inequalities within society.

As this metasynthesis also describe, girls examine their context and their peers (women and other girls), using it to compare themselves and set ideals, which influence their notions of well-being. Studies have shown that comparative thinking affects how girls see themselves and their bodies, and it is a relevant factor that influences girls’ psychological well-being, particularly during adolescence (Schutz et al., 2002). The authors found that girls self-evaluate themselves and opt for some behaviours (e.g., dieting, exercises) based on comparisons within different life domains either with peers or with ideals. This metasynthesis, particularly the studies from Europe and the U.S., shows how comparisons to ideals are detrimental to girls’ well-being. Girls compare themselves to other girls and to ideals, which impacts their well-being and plays an important role in the way they structure their social interactions. Through comparison, girls position themself within a hierarchy that determines the quality of their interactions and their possibilities of belonging with their group of peers. Discroll (2002) argued that in Western society, girls interpret their body image based on a larger discursive framework that involves formal education. Western schools include girls’ health education as it relates to diet and physical activity, which in turn shapes girls’ bodies towards an ideal image, massively promoted in media culture.

However, comparative thinking does not only include social and bodily comparisons, as it is in western society. It also includes opportunities and material resources, as Wilson and Somhlaba (2016) described. The inclusions of these other elements as fundamental for well-being is in line with African studies, in which significant insights on the relevance of material conditions and their fundamental influence in the experience of well-being are considered (Mahali et al., 2018). These studies challenge the normative approaches to well-being from the global North perspectives (Mahali et al., 2018; White, 2010). They ask for the inclusion of socio-economic aspects in the analysis of well-being (e.g., social capital, networks) and other identity axes (e.g., class, caste, religion) that are relevant to the global South. In that line, studies conducted in low-income countries have described children and adolescents compare themselves to others to define their socio-economic status, having a relative view of what is, for example, living in poverty, which is interwoven with relational aspects that highlighting exclusion as its fundamental consequence (Camfield, 2010).

### Study limitations

The current metasynthesis is not exempt from limitations. Being done by one author instead of a team of qualitative researchers, this metasynthesis may lack the richness of different perspectives and interpretations. As Thorne (2017) has remarked, metasynthesis are complex, and a team of researchers is desirable to increase the quality of the dialogue between the studies. This type of project is better suited for teams and would be interesting to compare results to other researchers to add more density to the analysis.

Secondly, there was a small number of studies that meet the inclusion criteria. A more flexible inclusion criteria may allow to include a larger number of studies. This would give more diversity to the results, as for example, works in Spanish or in Portuguese could be included. In addition, a different type of quality appraisal could allow to include less traditional qualitative studies, that follow different epistemological and methodological guidelines to examine adolescent girls’ well-being (e.g., poststructural and/or posthumanist studies).

### Future directions

This metasynthesis exposes that the qualitative study of adolescent well-being should advance on the recognition of the diversity of adolescent, generating contextualized knowledge that helps researchers to understand multilevel threats to well-being. Qualitative research on well-being should be recognized that age is intertwined with many other determinants that affect adolescents’ experiences, including other axes of identity. In that line, it is worrying that race as an analytical social category was absent from the selected studies, considering its undeniable impact on the human experience.

The results of this metasynthesis show that context and gender play a key role in the way well-being is experienced by adolescent girls. Qualitative studies on adolescent well-being may benefit from explicitly recognize gender as an analytical factor. An explicit gender perspective describing singularities and commonalities among adolescents is needed to advance the study and promotion of female adolescents’ well-being.

More qualitative research in different contexts is also needed to understand the cultural diversity of adolescent girls’ experiences. The limited geographical contexts included in this metasynthesis make apparent the need to conduct qualitative research on other geographical areas. It is presumable that qualitative knowledge of female adolescent well-being from different parts of the world would unveil different threats to well-being and injustices, as well as diverse experiences that may give better foundations to localized public policies.

### Conclusions

Qualitative research on adolescent girls’ well-being offers important insights to generate strategies for its promotion. Qualitative metasynthesis is a helpful approach to systematize, summarize and reinterpret qualitative knowledge since it provides better use of the existent research and identifies key elements that are singular to different contexts without reducing them to dominant narratives of well-being.

Better use of qualitative research in the study of well-being is also an ethical imperative. It illuminates girls’ experiences and opinions, and that knowledge can be used to guide and inform quantitative research, scale construction, or public policies, among others, from a contextualized perspective. As qualitative research can offer complex and illustrative insights on girls’ well-being, their potential should not be disregarded by those who design strategies to promote well-being.

As this metasynthesis shows, girls’ well-being is defined by factors at different levels, from family and immediate community influences to broader social and cultural practices. Consequently, interventions and public policies should study the key factors girls identified as influencing their well-being, and address them using a multilevel rationale. For example, strategies to ensure girls have equal access to resources and opportunities within families and communities should also include actions at broader societal levels, considering how sociocultural influences have set these types of practices as the norm. To promote supportive family and community environments that prevent exhausting expectations and demands directed towards girls, interventions must dismantle the gender norms that have upheld those demands. And while schools and families should avoid promoting comparative thinking between adolescent girls, their efforts are less likely to succeed if media messages continue to endorse them.

This metasynthesis has shown that deeply embedded in context are invisible and naturalized social practices. Those practices become recurring patterns of everyday interactions between girls and the environment in which they operate, affecting their well-being experiences. Understanding the powerful influence of contextual forces on girls’ well-being is fundamental to develop a relational and contextualized view of the phenomenon that supersede individual levels of analysis.

## References

[cit0001] Atkinson, S., Bagnall, A.M., Corcoran, R., South, J., Curtis, S., Di Martino, S., & Pilkington, G. (2017). What is Community Wellbeing? *Conceptual Review. What Works Wellbeing*. 10.13140/RG.2.2.29797.70889

[cit0002] Bintliff, A. V. (2019). *“And I just keep building myself up”: A grounded theory of the wellbeing of middle school girls with histories of family stressors participating in an after-school wellbeing club*. University of Wisconsin-Madison.

[cit0003] Camfield, L. (2010). “Stew without bread or bread without stew”: Children’s understandings of poverty in Ethiopia. *Children and Society*, 24(4), 271–14. 10.1111/j.1099-0860.2010.00311.x

[cit0004] Camfield, L., Crivello, G., & Woodhead, M. (2009). Wellbeing research in developing countries: Reviewing the role of qualitative methods. *Social Indicators Research*, 90(1), 5–31. 10.1007/s11205-008-9310-z

[cit0005] Casas, F., Bello, A., González, M., & Aligué, M. (2013). Children’s subjective well-being measured using a composite index: What impacts spanish first-year secondary education students’ subjective well-being? *Child Indicators Research*, 6(3), 433–460. 10.1007/s12187-013-9182-x

[cit0006] Cooke, A., Smith, D., & Booth, A. (2012). Beyond PICO: The SPIDER tool for qualitative evidence synthesis. *Qualitative Health Research*, 22(10), 1435–1443. 10.1177/1049732312452938 22829486

[cit0007] Cosgrove, L. (2003). Feminism and psychological research. *Hypatia*, 18(3), 85–112. 10.1111/j.1527-2001.2003.tb00823.x

[cit0008] Critical Appraisal Skills Programme. (2018). *CASP qualitative checklist*. https://casp-uk.net/wp-content/uploads/2018/01/CASP-Qualitative-Checklist-2018.pdf

[cit0009] Diener, E., Heintzelman, S. J., Kushlev, K., Tay, L., Wirtz, D., Lutes, L. D., & Oishi, S. (2017). Findings all psychologist should know from the new science on subjective well-being. *Canadian Psychology/Psychologie Canadienne*, 58(2), 87–104. 10.1037/cap0000063

[cit0010] Driscoll, C. (2002) Girls: Feminine adolescence in popular culture and cultural theory. Columbia University Press

[cit0011] Evans, S. (2014). Well-being. In T. Teo (Ed.), *Encyclopedia of critical psychology* (pp. 2073–2075). Springer. 10.1007/978-1-4614-5583-7

[cit0012] Evans, S., & Prilleltensky, I. (2007). Youth and democracy: Participation for personal, relational, and collective well-being. *Journal of Community Psychology*, 35(6), 681–692. 10.1002/jcop.20172

[cit0013] Fattore, T., Fegter, S., & Hunner-Kreisel, C. (2019). Children’s understandings of well-being in global and local contexts: Theoretical and methodological considerations for a multinational qualitative study. *Child Indicators Research*, 12(2), 385–407. 10.1007/s12187-018-9594-8

[cit0014] Finfgeld, D. L. (2003). Metasynthesis: The state of the art - So far. *Qualitative Health Research*, 13(7), 893–904. 10.1177/1049732303253462 14502956

[cit0015] Finfgeld-Connett, D. (2018). *A guide to qualitative meta-synthesis*. Taylor & Francis. 10.4324/9781351212793

[cit0016] Flacking, R., Jerdén, L., Bergström, E., & Starrin, B. (2014). ‘In or out’—On the dynamic between acceptance and rejection and its influence on health in adolescent girls. *Young*, 22(3), 291–303. 10.1177/1103308814534043

[cit0017] Flook, L. (2011). Gender differences in adolescents ’ daily interpersonal events and well-being. *Child Development*, 82(2), 454–461. 10.1111/j.l467-8624.2010.01521.x 21410907

[cit0018] Glozah, F. N. (2015). Exploring Ghanaian adolescents’ meaning of health and wellbeing: A psychosocial perspective. *International Journal of Qualitative Studies on Health and Well-Being*, 10(1), 26370. 10.3402/qhw.v10.26370 25855158 PMC4390561

[cit0019] González-Carrasco, M., Vaqué, C., Malo, S., Crous, G., Casas, F., & Figuer, C. (2019). A qualitative longitudinal study on the well-being of children and adolescents. *Child Indicators Research*, 12(2), 479–499. 10.1007/s12187-018-9534-7

[cit0020] Gough, I., McGregor, A., & Camfield, L. (2007). Theorising wellbeing in international development. In I. Gough & J. A. McGregor (Eds.), *Wellbeing in Developing Countries* (Vol. 9780521857). Cambridge University Press. 10.1017/CBO9780511488986

[cit0021] Gross-Manos, D., & Ben-Arieh, A. (2017). How subjective well-being is associated with material deprivation and social exclusion in Israeli 12-year-olds. *American Journal of Orthopsychiatry*, 87(3), 274–290. 10.1037/ort0000160 26986840

[cit0022] Helseth, S., & Misvær, N. (2010). Adolescents’ perceptions of quality of life: What it is and what matters. *Journal of Clinical Nursing*, 19(9–10), 1454–1461. 10.1111/j.1365-2702.2009.03069.x 20500355

[cit0023] Igarashi, Y. (2015). Health psychology: Towards critical psychologies for well-being and social justice. In I. Parker (Ed.), *Handbook of critical psychology* (pp. 173–181, Issue 2015). Taylor & Francis.

[cit0024] Larsson, M., Sundler, A. J., & Ekebergh, M. (2013). Beyond self-rated health: The adolescent girl’s lived experience of health in Sweden. *Journal of School Nursing*, 29(1), 71–79. 10.1177/1059840512446151 22550164

[cit0025] Levy, J. K., Darmstadt, G. L., Ashby, C., Quandt, M., Halsey, E., Nagar, A., & Greene, M. E. (2020). Characteristics of successful programmes targeting gender inequality and restrictive gender norms for the health and wellbeing of children, adolescents, and young adults: A systematic review. *The Lancet Global Health*, 8(2), e225–e236. 10.1016/S2214-109X(19)30495-4 31879212 PMC7025324

[cit0026] Mahali, A., Lynch, I., Fadiji, A. W., Tolla, T., Khumalo, S., & Naicker, S. (2018). Networks of well-being in the Global South: A critical review of current scholarship. *Journal of Developing Societies*, 34(4), 373–400. 10.1177/0169796X18786137

[cit0027] Mattos, A. (2015). Feminist psychology: Reserach interventions, challenges. In I. Parker (Ed.), *Handbook of critical psychology* (pp. 329–338). Routledge. 10.4324/9781315726526.ch34

[cit0028] Methley, A. M., Campbell, S., Chew-Graham, C., McNally, R., & Cheraghi-Sohi, S. (2014). PICO, PICOS and SPIDER: A comparison study of specificity and sensitivity in three search tools for qualitative systematic reviews. *BMC Health Services Research*, 14(1). 10.1186/s12913-014-0579-0 PMC4310146 25413154

[cit0029] Miller, J. B. (1976). *Toward a new psychology of women*. Beacon Press.

[cit0030] Molony, S. L. (2010). The meaning of home: A qualitative metasynthesis. Research in Gerontological Nursing,3(4), 291–307. 10.3928/19404921-20100302-02 20429493

[cit0031] Montserrat, C., Dinisman, T., Bălţătescu, S., Grigoraş, B. A., & Casas, F. (2015). The effect of critical changes and gender on adolescents: Subjective well-being: Comparisons across 8 countries. *Child Indicators Research*, 8(1), 111–131. 10.1007/s12187-014-9288-9

[cit0032] Newton, J., & Ponting, C. (2013). Eliciting young people’s views on wellbeing through contemporary science debates in Wales. *Child Indicators Research*, 6(1), 71–95. 10.1007/s12187-012-9159-1

[cit0033] Phiri, D. T., & Abebe, T. (2016). Suffering and thriving: Children’s perspectives and interpretations of poverty and well-being in rural Zambia. *Childhood*, 23(3), 378–393. 10.1177/0907568216637654

[cit0034] Prilleltensky, I. (2012). Wellness as fairness. *American Journal of Community Psychology*, 49(1–2), 1–21. 10.1007/s10464-011-9448-8 21643926

[cit0035] Ronen, T., Hamama, L., Rosenbaum, M., & Mishely-Yarlap, A. (2016). Subjective well-being in adolescence: The role of self-control, social support, age, gender, and familial crisis. *Journal of Happiness Studies*, 17(1), 81–104. 10.1007/s10902-014-9585-5

[cit0036] Saldaña., J. (2013). Coding manual. Sage

[cit0037] Sandelowski, M., & Barroso, J. (2007). *Handbook for synthesizing qualitative research*. Springer Publishing Company.

[cit0038] Savahl, S., Malcolm, C., Slembrouk, S., Adams, S., Willenberg, I. A., & September, R. (2015). Discourses on well-being. *Child Indicators Research*, 8(4), 747–766. 10.1007/s12187-014-9272-4

[cit0039] Schutz, H. K., Paxton, S. J., & Wertheim, E. H. (2002). Investigation of body comparison among adolescent girls. *Journal of Applied Social Psychology*, 32(9), 1906–1937. 10.1111/j.1559-1816.2002.tb00264.x

[cit0040] Seligman, M. E. P., & Csikszentmihalyi, M. (2014). Positive psychology: An introduction. *Flow and the Foundations of Positive Psychology*, 55(1), 279–298. 10.1037/0003-066X.55.1.5 11392865

[cit0041] Skrzypiec, G., & Askell-Williams, H. (2018). Girls’ diminishing wellbeing across the adolescent years. In P. Slee, G. Skrzypiec, & C. Cefai (Eds.), *Child and adolescent wellbeing and violence prevention in schools* (pp. 60–69). Oxon. 10.4324/9781315102047-7

[cit0042] The Global Women’s Institute at The George Washington University. (2019, 2). *EVIDENCE BRIEF school-based interventions to prevent violence against women & girls*. https://globalwomensinstitute.gwu.edu/sites/g/files/zaxdzs1356/f/downloads/Evidence%20Brief-%20School-Based%20Interventions%20to%20Prevent%20Violence%20Against%20Women%20and%20Girls.pdf

[cit0043] Thorne, S. (2017). Metasynthetic madness: What kind of monster have we created? *Qualitative Health Research*, 27(1), 3–12. 10.1177/1049732316679370 27956657 PMC5154391

[cit0044] UNICEF, UN Women, & Plan International. (2020). *A new era for girls. Taking stock of 25 years of progress*. UNICEF. www.unicef.org/gender

[cit0045] Vujčić, M. T., Brajša-Žganec, A., & Franc, R. (2019). Children and young peoples’ views on well-being: A qualitative study. *Child Indicators Research*, 12(3), 791–819. 10.1007/s12187-018-9559-y

[cit0046] White, S. C. (2010). Analysing wellbeing: A framework for development practice. *Development in Practice*, 20(2), 158–172. 10.1080/09614520903564199

[cit0047] White, S. C. (2017). Relational wellbeing: Re-centring the politics of happiness, policy and the self. *Policy & Politics*, 45(2), 121–136. 10.1332/030557317X14866576265970

[cit0048] White, S. C., & Blackmore, C. (2015). *Cultures of wellbeing: Method, place, policy* (S. C. White & C. Blackmore (eds.)). Palgrave Macmillan UK. 10.1057/9781137536457

[cit0049] Wiens, V., Kyngäs, H., & Pölkki, T. (2014). A descriptive qualitative study of adolescent girls’ well-being in Northern Finland. *International Journal of Circumpolar Health*, 73(1), 24792. 10.3402/ijch.v73.24792 25317384 PMC4185135

[cit0050] Wiens, V., Kyngäs, H., & Pölkki, T. (2017). Issues promoting and hindering girls’ well-being in Northern Finland. *Health Promotion International*, 32(4), 671–680. 10.1093/heapro/daw006 26902099

[cit0051] Wigginton, B., & Lafrance, M. N. (2019). Learning critical feminist research: A brief introduction to feminist epistemologies and methodologies. *Feminism and Psychology*, 0(0), 1–17. 10.1177/0959353519866058

[cit0052] Wilson, A., & Somhlaba, N. Z. (2016). Psychological well-being in a context of adversity: Ghanaian adolescents’ experiences of hope and life satisfaction. *Africa Today*, 63(1), 85–103. 10.2979/africatoday.63.1.0085

[cit0053] Wodon, Q., Montenegro, C., Nguyen, H., & Onagoruwa, A. (2018) *Missed Opportunities: the high cost of not educating girls (The Cost of Not Educating Girls Notes Series, Issue July)*.

[cit0054] Wright, K., & Mcleod, J. (2015). *Rethinking youth wellbeing* (K. Wright & J. McLeod (eds.)). Springer Singapore. 10.1007/978-981-287-188-6

[cit0055] Yin Nei Cho, E. (2018). Links between poverty and children’s subjective wellbeing: Examining the mediating and moderating role of relationships. *Child Indicators Research*, 11(2), 585–607. 10.1007/s12187-017-9453-z

